# Bacteria and tumours: causative agents or opportunistic inhabitants?

**DOI:** 10.1186/1750-9378-8-11

**Published:** 2013-03-28

**Authors:** Joanne Cummins, Mark Tangney

**Affiliations:** 1 Cork Cancer Research Centre, BioSciences Institute, University College Cork, Cork, Ireland

## Abstract

Associations between different bacteria and various tumours have been reported in patients for decades. Studies involving characterisation of bacteria within tumour tissues have traditionally been in the context of tumourigenesis as a result of bacterial presence within healthy tissues, and in general, dogma holds that such bacteria are causative agents of malignancy (directly or indirectly). While evidence suggests that this may be the case for certain tumour types and bacterial species, it is plausible that in many cases, clinical observations of bacteria within tumours arise from spontaneous infection of established tumours. Indeed, growth of bacteria specifically within tumours following deliberate systemic administration has been demonstrated for numerous bacterial species at preclinical and clinical levels. We present the available data on links between bacteria and tumours, and propose that besides the few instances in which pathogens are playing a pathogenic role in cancer, in many instances, the prevalent relationship between solid tumours and bacteria is opportunistic rather than causative, and discuss opportunities for exploiting tumour-specific bacterial growth for cancer treatment.

## Introduction

The development of cancer is associated with several genetic and environmental factors. Furthermore there has been an association between the development of cancer and bacterial and viral infections for decades. Several viruses can integrate into the human genome and directly initiate tumourigenesis, such as human papillomavirus (HPV) in cervical cancer and herpesvirus in Kaposi’s sarcoma [1,2]. In other cases, the development of cancer is indirect, such as with *Helicobacter pylori*, which contributes to both gastric cancer and mucosa-associated lymphoid tissue (MALT) lymphoma due to chronic inflammation caused by the bacteria [3,4]. This notwithstanding, observations made in the course of numerous studies have reported different indigenous bacterial species being isolated from infected lesions in patients (Table 1) [5,6]. As early as 1868 Bush reported 2 patients with sarcoma that had been infected with *Streptococcus* and Coley also detected the presence of *S. pyogenes* in a patient suffering from neck cancer [7,8]. In 1907 Fibiger discovered that the parasitic nematode *(Spiroptera neoplastica*) was associated with cancer development in rats, while it was later found that the development of cancer was associated with a vitamin A deficiency he paved the way for future discoveries in cancer causing bacteria and viruses [9]. In 1911 Francis Peyton Rous was the first to discover that cancer could be transmitted by a virus (later known as Rous sarcoma virus), but it wasn’t until years later that his results were accepted by the scientific community [10,11]. In 1926 Glover stated that certain bacteria were consistently isolated from neoplastic tissue [12]. Furthermore between the years of 1936–1955 several different publications all reported the presence of microbes in cancer tissue [13-15]. More recently, it has been shown that bacteria are naturally capable of homing to tumours when systemically administered, resulting in high levels of replication locally [16]. This was originally established following IV administration of species of *Clostridium*, and in more recent years with numerous bacterial species, including*, Salmonella, Bifidobacterium, Escherichia coli*, *Vibrio cholerae* and *Listeria monocytogenes*. Furthermore, various clinical trials have shown the ability of different bacterial strains to home to and replicate specifically within tumours [17-19].

**Table 1:** Overview of bacteria identified in different tumour types

**Cancer**	**Cancer role**	**Reference**
**Lung cancer**		
*Streptococcus mitis*	Prevalent	[20]
*Staphylococcus epidermis*	Prevalent	[20]
*Bacillus* sp.	Prevalent	[20]
*Mycoplasma* sp.	Causative	[20,21]
*Chlamydophila pneumonia*	Causative	[22]
**Pancreatic cancer**		
*Robinsoniella peoriensis*	Prevalent	[23]
*Pedioccoccus acidilactici*	Prevalent	[24]
*Leuconostoc lactis*	Prevalent	[24]
*L. mesenteroides*	Prevalent	[24]
**Breast cancer**		
*Staphylococcus epidermidis*	Prevalent	[25]
*Mycoplasma* sp.	Prevalent	[21]
**Oral cancer**		
*Ralstonia insidiosa*	Prevalent	[26]
*Fusobacterium naviforme*	Prevalent	[26]
*Prevotella* sp.	Prevalent	[26]
**Gall-bladder carcinoma**		
*Salmonella typhi*	Causative	[27]
*H. pylori*	Causative	[28]
*H. hepaticus*	Causative	[28]
*H. bilis*	Causative	[29]
**Pulmonary Mucosa-Associated lymphoid tissue (MALT) lymphoma**		
*Chlamydia pneumonia*	Causative	[30]
*C. trachomatis*	Causative	[30]
*C. psittaci*	Causative	[30]
**Ocular Adenexa MALT lymphoma**		
*C. psittaci*	Causative	[31]
**Ovarian cancer**		
*Chlamydia trachomatis*	Prevalent	[32]
*Mycoplasma* sp.	Causative	[33]
*Mycoplasma* sp.	Causative	[21]
**Colorectal cancer**		
*Streptococcus gallolyticus*	Causative	[34]
*Fusobacterium nucleatum*	Prevalent (?)	[35,36]
*F. necrophorum*	Prevalent	[36,37]
*F. mortiferum*	Prevalent	[36]
*F. perfoetens*	Prevalent	[36]
*Roseburia* sp.	Prevalent	[37]
*Faecalibacterium* sp.	Prevalent	[37]
*Escherichia coli*	Prevalent	[38]
*Citrobacter* sp.	Prevalent	[38]
*H. pylori*	Causative	[39]
*Mycoplasma* sp.	Prevalent	[21]

The presence of bacteria within tumours could be due to infection via the vasculature and their ability to survive and grow due to the presence of nutrients within the hypoxic region of the tumour at a later stage in tumour growth (see below for proposed mechanism). For example, sampling from humans has indicated that bacterial translocation of Gastro-Intestinal Tract (GIT)-associated bacteria may be a phenomenon that occurs in healthy individuals representing a normal physiological event without deleterious consequences [40]. It may be that bacteria egress from the GIT at very low numbers, and are normally quickly eliminated by the immune system. However, the phenomenon of bacterial replication within tumours results in dramatic increases in bacterial numbers within a confined region.

This review will focus on bacteria that have been identified within patient tumours and how they might be used to aid in early detection (in the context of causative agents) or treatment (subsequent infection) of cancers.

### Bacteria as causative agents of cancer

There are several bacteria that have been associated or defined as being causative agents of cancer (Table 1). The most widely known of these bacteria would be *H. pylori* which is the strongest known risk factor for gastric cancer [41]. However only a small minority of those infected develop gastric cancer or precancerous gastric lesions. The reason for this is thought to be due to several factors; strain biodiversity, geographical distribution and environmental factors. *H*. *pylori* is also associated with an increased risk of pancreatic cancer, thought to occur through pathophysiological actions of the bacterium [41,42]. Recent data also support the hypothesis that *H. pylori* is also associated with a slightly increased risk of colorectal cancer [39].

Furthermore recent data promotes the paradigm that gut bacteria can influence cancer risk in extra-intestinal organs [43,44]. The means by which this occurs is not fully elucidated, but is thought to involve both immunity and metabolism as key factors in tumour promotion by intestinal bacteria [45]. *H. hepaticus* is a murine enterohepatic bacterium that causes hepatitis and hepatocellular carcinoma (HCC) in some strains of mice [46]. A recent study by Rogers demonstrated increased liver tumours in mice colonized with *H. hepaticus* in the lower bowels but without any requirements for hepatic translocation or hepatitis induction [45]. This lead to the conclusion, that *H. hepaticus* promotes liver cancer from its endogenous niche in the lower bowels. Another study using Apc^min/+^Rag2^−/−^ mice demonstrated an increased risk of mammary cancer when infected with *H. hepaticus*, further suggesting that gut microbes can promote extra-intestinal cancer [44].

One of the bacterial agents that have been found to be regularly associated with colorectal carcinoma (CRC) is *Streptococcus bovis/gallolyticus*. *S. bovis/gallolyticus* is a transient normal flora in the gut thought to be present in 2.5-15% of individuals [47-49]. One theory for the association between *S. bovis/gallolyticus* and CRC is due the increased load of *S. bovis/gallolyticus* in the colon, which has been shown to be associated with inflammatory bowel disease or malignant/premalignant lesions of the tumour. From the research preformed to date it is believed that *S. bovis/gallolyticus* association with CRC seems to be of etiological nature and the proposed carcinogenic potential of *S. bovis/gallolyticus* is most likely a propagation factor for premalignant tissues. The early detection of CRC via identification of *S. bovis/gallolyticus* DNA or antibodies may be a potential screening method for at high-risk groups [50].

*C. pneumoniae* is a Gram-negative bacillus and an obligate intracellular parasite that causes respiratory infections in over 50% of adults. The relationship between *C. pneumoniae* and lung cancer has been studied for over 10 years by clinical and laboratory research methods but the results have been inconsistent. Recently a meta-analysis approach was used to analyse previously published data [51]. They concluded a dose–response effect in which increasing lung cancer risk was associated with increasing IgA (serological criteria for chronic infection) antibody titre, also suggested that higher titre may be a better predictor of lung cancer risk than lower antibody titres [51]. All these studies also indicated that *C. pneumoniae* infection was associated with increased risk of lung cancer in certain sub-groups such as young individuals, men, former smokers and for squamous cell carcinomas or small cell carcinomas [22,51-55].

### The other side of the coin: opportunistic infections of established tumours

While bacterial presence in certain tumours is associated with the development of that cancer, in many cases, bacteria present in tumours may reflect local infections of existing malignant tissue [56]. Observations made in the course of numerous studies have reported different indigenous bacterial species being isolated from infected lesions in patients (Table 1). After Bush’s report of *Streptococcus* in sarcomas in 1868 [7], William Coley, the “father of immunotherapy”, identified the presence of *S. pyogenes* in soft tissue sarcoma, and was one of the first to characterise concomitant infection of tumours and how this could lead to remission of incurable neoplastic malignancy [57,58]. In recent years there have been a number of studies that show a correlation between bacterial infection and tumour regression. Ruckdeschel *et al.* found that patients who developed empyema after lung cancer had a significantly improved survival rate after 5 years compared with uninfected patients (50% v’s 18%) [59]. Furthermore, two patients with malignant CNS tumours unexpectedly regressed after infection. Of these patients, one of the infections was viral in nature while the second was found to feature *Corynebacterium hemolyticum* and *Staphylococcus epidermidis*. The patient survived for 9 years after his tumour was originally discovered [60]. A recent study to determine the survival rate after infection in glioblastoma multiforme patients demonstrated no survival advantage in patients with post-operative infection but they did find that the deep infection subgroup showed a trend towards increased survival [61]. Bohman and colleagues literature search revealed 9 patients with malignant intracranial tumours who experienced prolonged survival due to infection with a mean survival of 7.5-8 years and no evidence of central nervous system recurrence upon death. The majority of patients had infections with either *S. aureus* or *Enterobacter aerogens*[61].

#### Mechanisms of bacterial replication within tumours

There are several proposed factors involved in how bacteria replicate and survive within tumours. Traditionally, the main mechanism is thought to be due to the hypoxic nature of many solid tumours, which results in low oxygen levels compared with normal tissues, providing a unique growth environment for anaerobic and facultative anaerobic bacteria [62]. Other factors contributing to bacterial replication in the tumour include the presence of bacterial nutrients within the necrotic region such as purines [63]. Furthermore, the involvement of bacterial chemotaxis towards chemo-attractant compounds present in necrotic regions (e.g. aspartate, serine, citrate, ribose or galactose) produced by quiescent cancer cells has also been suggested as a contributing factor [63].

**Figure 1:**
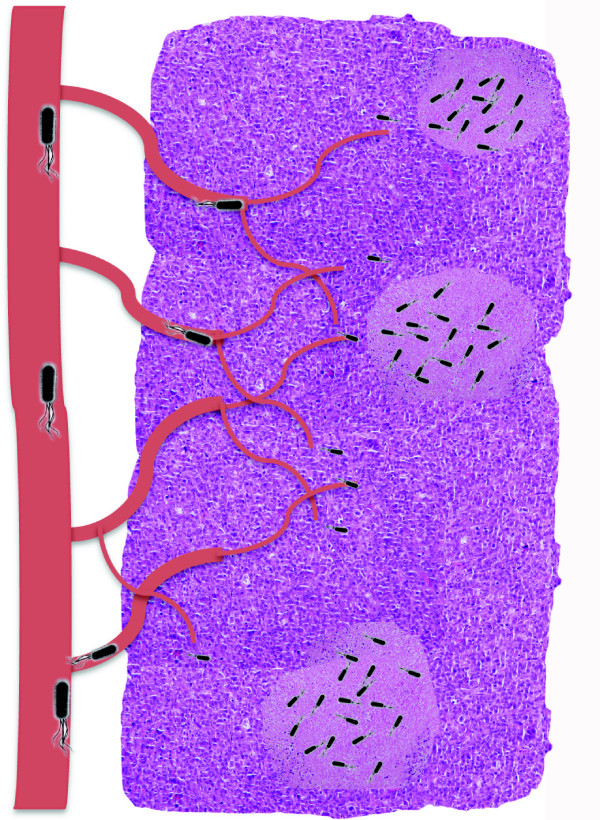
**Proposed mechanism of bacterial entry to and proliferation within tumours.** Tumour (pink/purple) development leads to recruitment of new blood supply, involving disorganized and leaky vasculature, permitting circulating bacteria (black) to enter the tumour. Bacteria replicate primarily within hypoxic (pink) tumour regions, which feature immune suppression, abundant nutrients and low oxygen.

As knowledge in the field grows, elements believed to be key for tumour-specific bacterial replication include aberrant neovasculature and local immune suppression [63]. As tumours develop, they promote the construction of new blood vessels (neo-angiogenesis). However, these newly formed vessels are highly disorganized with incomplete endothelial linings and blind ends, resulting in ‘leaky’ blood vessels and slow blood flow. This leaky tumour vasculature may allow circulating bacteria to enter tumour tissue, and embed locally (Figure 1) [63]. Furthermore, a variety of mechanisms are employed by cancerous cells to avoid recognition by the immune system resulting in insufficient immune activity within tumours, potentially providing a refuge for bacteria to evade immune clearance, not present elsewhere in the body [64,65].

#### Systemic microbes: routes of infection

While blood is taken to be free of bacteria in healthy individuals, it is plausible that establishment of a tumour infection might occur even with extremely low numbers of viable bacteria, given the phenomenon of tumour-specific bacterial growth. This hypothesis suggests the tumour providing a localized ‘amplified read-out’ for bacterial infection, which is otherwise unapparent. Presence of bacteria in the blood stream may be due to wound infection (e.g. post surgery), or bacterial translocation (BT) from the gastrointestinal tract (GIT).

#### GIT translocation

BT is the invasion of indigenous intestinal bacteria through the gut mucosa into normal sterile tissue [66]. The means by which bacteria translocate is thought to be by two distinct pathways of gastrointestinal permeability: (1) transcellular through the enterocytes and (2) paracellular using tight junctions [67]. There are also thought to be two major methods by which bacterial compounds gain access to systemic circulation; (1) through the enteric venous system to the portal vein and (2) following lymphatic drainage. After much analysis it is thought that the lymphatic route is the primary pathway of translocation [67]. Experimental and clinical studies have detected both indigenous and non-indigenous bacteria within the mesenteric lymph node (MLN). Some studies have demonstrated that BT from the gut to the MLN is quiet common, with it occurring in 4-59% of patients [67]. Furthermore patients needing surgery for abdominal infection fared much worse if the MLN was infected with non-indigenous bacteria [67]. In normal healthy individuals, the normal immune response will destroy bacteria associated with BT by phagocytosis. However, immunocompromised individuals have a higher risk of being affected by BT. Cannon *et al.*, found that one of the main underlying conditions increasing patients susceptibility to BT associated bacteraemia was cancer [68]. Penn and colleagues demonstrated an increased translocation from the GIT of S-180 tumour-bearing mice, leading them to postulate that the immune deficiencies associated with tumour growth may be adequate to allow the viable bacteria to translocate from the GIT to the developing tumour [69]. Our group has shown that oral administration of non-pathogenic bacteria to mice results in trafficking from the GIT with subsequent homing to and replication specifically in tumours [70].

The ability of microorganisms to translocate, survive, and proliferate in extra-intestinal tissues involves complex interactions between the host defence mechanisms and the bacterium’s ability to invade host tissues. Although the importance of host immune function and the bacterium’s intestinal population size have been implicated as significant contributory factors, the precise mechanisms involved remains unknown [5,66,69,71].

#### The oral microbiome

As described earlier, members of the oral microbiota have been associated with pancreatic cancer [72,73]. Farrell and colleagues carried out microbial profiling using the Human Oral Microbe Identification Microarray (HOMIM) to compare the salivary microbiota between 10 pancreatic cancer patients and 10 normal matched healthy controls [72]. In their study they found that the levels of *Neisseria elongata* and *Streptococcus mitis* were decreased in the cancer patients relative to healthy controls and the level of *Granulicaetella adiacens* was increased in the cancer patients. These results validate a link between *N. elongata* and *G. adiacens* with periodontal disease, which has been linked to an increased risk of pancreatic cancer [72]. Farrell *et al.*, postulated that as *G. adiacens*, an opportunistic pathogen, may be associated with systemic inflammation and an elevation in this bacteria could be related to the decreased levels of *S. mitis*[72]. Furthermore *S. mitis* plays a protective role against the adhesion of carcinogenic bacteria and the loss of *S. mitis* may contribute to aggressive periodontitis. Whether the variation in oral bacteria in pancreatic cancer is causative rather than reactive would have to be investigated further to determine how local oral bacteria without entering the blood stream could potentially cause systemic diseases, chronic inflammation and neoplasia [72]. In the case of periodontal bacteria, such as *Porphyromonas gingivalis* it is believed that lymph vessel openings trap bacteria en route from the mouth to the blood stream and then carry them to the vein of the venous angle near the supraclavicular area [74]. A previous study by Sakamoto and colleagues analysed the regional lymph nodes of 30 patients with oral cancer [75]. A total of 153 lymph nodes were harvested for microbiological examination. It was found that viable bacteria were found in 45% of the lymph nodes from 83% of patients. The cervical lymph nodes are in contact with the lymphatic flow before returning to the thoracic duct. The presence of viable bacteria, mainly oral streptococcus, in these lymph nodes supports the hypothesis of the association between oral mucosal damage and bacterial invasion into general circulation [75]. Furthermore the anaerobic bacteria detected (*Peptostreptococcus*) in the lymph nodes have been detected in gastrointestinal tumour tissue suggesting that the oral mucosal defect could promote translocation of anaerobes into the regional nodes [75,76].

Farrell and colleagues then evaluated the specificity of the microbial biomarkers against another HOMIM microbial study that had been performed on lung cancer. They observed that none of the bacterial biomarkers validated in their study was significantly altered in the microflora profile of lung cancer [72]. This validates that the microbial biomarkers present in the saliva are specific for pancreatic cancer.

#### Fusobacterium

A bacterium that of late has been linked with CRC is *Fusobacterium nucleatum*[35,36]. *F. nucleatum* is an invasive, adherent and pro-inflammatory anaerobe that is commonly found in dental plaque and is well known to be associated with periodontitis [77,78,79]. However, *Fusobacterium* is also known to be a gut commensal with probiotic features [79]. Castellarin and colleagues analysed the RNA sequence of 11 matched pairs of colorectal carcinoma and adjacent normal tissue and found an over-representation of *F. nucleatum*[35]. They postulated that the presence of the bacterium within the tumour could just represent an prevalent infection at an immunocompromised site or may be involved in tumourigenesis [75].

Another study using a similar method identified the presence of several *Fusobacterium* species in CRC, in particular *F. nucleatum*, *F. mortiferum* and *F. necrophrum*[36]. They believe that *Fusobacterium* may be following the concept of the “alpha-bug” theory –where certain members of a microbial community are capable of remodelling the microbiome as a whole to drive proinflammatory immune responses and colonic epithelial cell transformation leading to cancer [36,80]. In addition, *Fusobacterium* has been associated with inflammatory bowel disease (IBD), one of the three highest risk factors for developing CRC [81]. Kostic and colleagues postulate that *Fusobacterium* may contribute to tumourigenesis in a limited subset of patients mainly thorough an inflammatory mediated mechanism [36].

One interesting facet of *F. nucleatum* is that it is one of the most prevalent species found in infections of the amniotic fluid and the placenta leading to pre-term birth [82,83]. There is also a correlation between pre-term birth and periodontal disease. During periodontal infection the quantities of periodontal pathogens increases dramatically leading to transient bacteraemia [84-86]. This can lead to selective colonization of undesired sites. The ability of *F. nucleatum* to proliferate in the placenta and eventually spread to the amniotic fluid and foetuses may in part be due to local immunosuppression in reproductive organs during pregnancy [87]. Furthermore the slow blood flow rate and low shear force in the venous sinuses of the placenta provides an opportunity for *F. nucleatum* to adhere and invade the endothelial cells [87]. As similar conditions occur within a tumour this may be a reason why *F. nucleatum* has been identified within CRC. Even if there is no etiological link between the bacterium and CRC the significant abundance of *Fusobacterium* in CRC may be used for screening purpose [35].

### Exploitation of opportunistic infective agents

The ideal anti-cancer therapy would selectively eradicate tumours, whilst minimizing side effects to normal tissue. Use of bacteria to specify therapeutic agents to tumours presents an attractive strategy in this context, since we and others have shown in mice that bacteria naturally replicate specifically within tumours when systemically administered [88]. Various preclinical trials have shown the ability of different bacterial strains to traffic to tumour sites, locally produce therapeutic agents, and mediate highly effective and specific therapeutic responses. Bacterial cancer therapies to date have utilized ‘laboratory’ strains (e.g. *Salmonella typhimurium* or clostridia), and while results in murine models have been impressive, successes have failed to be replicated in patients, with the inherent pathogenicity and immunogenicity of the bacteria employed outweighing therapeutic responses in patients.

Various preclinical and clinical trials have shown the ability of different bacterial strains to selectively traffic to tumour sites, and mediate highly effective and specific therapeutic responses [63]. A wide range of gene therapy strategies exists, aiming at inducing malignant cell death, either directly (e.g. using ‘suicide’ genes) or indirectly, such as cancer immunotherapy approaches based on killing tumour cells through intervention of various effector cells of the immune system [89]. The specific nature of bacterial colonisation of tumours could be exploited to aid in cancer treatment. In the case of non-invasive bacteria, strains can be engineered to secrete therapeutic proteins locally within the tumour environment, external to tumour cells [90]. This cell therapy approach is suitable for indirectly acting therapeutic strategies such as anti-angiogenesis and immune therapy.

Angiogenesis is the formation of new capillary blood vessels from existing microvessels [63]. The anti-angiogenesis strategy seeks to prevent the formation of new vessels. Gene-based anti-angiogenic therapy has been used in conjunction with other approaches to decrease angiogenesis. Bifidobacterial expression of endostatin genes, an endogenous inhibitor of angiogenesis, have shown promise in pre-clinical trials [63]. In addition *Salmonell*a VPN2009 has been successful in mediating anti-angiogenic therapy [63]. The immune therapy approach focuses on killing the tumour cell through direct or indirect alteration of immune effector cells (i.e. CD8^+^, T cells or NK cells). *S.* Typhimurium has been used in several murine trials examining immunotherapies, with significant tumour reduction resulting from local bacterial expression or tumour cell expression of the immune-stimulating molecules IL-18, CCL21, LIGHT or Fas ligand [63]. Preclinical studies have also used bifidobacteria in combination therapy with cytokines such as granulocyte colony-stimulating factor (GCSF), resulting in superior anti-tumour effects [63].

However, bacterial studies have largely focused on delivery of genes for subsequent tumour cell expression of anti-cancer agents utilizing pathogenic invasive bacterial species. Invasive bacteria are capable of delivering genes intracellularly with the aim of targeting bactofection to tumours. Bactofection is bacterial-mediated transfer of plasmid DNA to mammalian cells and has shown potential as a method to express heterologous proteins in different mammalian cells types [91,92]. In bactofection the bacteria contain a plasmid-based gene for transfer to the new host cells. Delivery of genetic material is achieved through entry of the entire bacterium into the target cells. Spontaneous or induced bacterial lysis leads to release of the plasmid for future eukaryotic cell expression. Various bacterial species including *Salmonella* spp*.*, *L. monocytogenes,* and *E. coli* have been examined as bactofection vectors. *Salmonella* is the most widely studied genus for bactofection with numerous studies demonstrating therapeutic expression and anti-tumour efficacy [63]. Preclinical trials utilising various attenuated, replication incompetent strains of salmonellae delivered by direct intratumoural or systemic administration have achieved impressive anti-tumour responses using a range of therapeutic approaches. The mechanism of DNA transfer is at present poorly understood for many species, and may depend on properties inherent to the bacteria and the cell type involved [93]. Invasive strains are pathogenic in nature and therefore invade healthy tissue (liver, spleen, MLN etc.), and safety concerns need to be addressed before such an approach becomes applicable in humans [63].

Furthermore bacteria, which replicate within tumours, can be engineered to express imaging agents, allowing detection of the bacteria and the potential tumour site [63]. Given the vital aspect of early detection for cancer treatment the potential for tumour-specific bacteria in diagnostic applications is attractive. If there is a definitive link between cancer development and bacterial infection this can be used in developing new treatment strategies for cancer. Furthermore microbes can affect the metabolism of pharmaceutical agents including those used to treat cancer. Therefore better knowledge of microbe composition and metabolic activity could improve therapeutic options.

## Conclusion

The examples provided here are not comprehensive, but are rather an indication of the alternative impacts bacteria may have on different types of cancers. Screening for bacteria present in cancer tissues of various histological types may open up new dimensions in our understanding of this relationship, and its importance, if any. As high throughput deep sequencing technologies become more available, mining for bacterial strains adapted to survive within the tumour microenvironment will permit dedicated studies on this phenomenon, perhaps even leading to the characterisation of a potential ‘tumour microbiome’, and may also advance the development of live bacterial vectors for tumour-specific delivery of therapeutic agents.

## Competing interests

The authors declare that they have no competing interests.

## Authors’ contributions

Both authors were involved in writing and reviewing this article. Both authors read and approved the final manuscript.
